# Enhancing cadaver preparation protocol to reduce bacterial contamination in musculoskeletal allografts: a comparative study of battlefield fatalities and general population donors

**DOI:** 10.1007/s10561-026-10208-4

**Published:** 2026-02-25

**Authors:** Helit Cohen, Moti Harats, Ron Burshtein, Alina Levi, Daniel Dothan, Marina BenShoshan, Michelle Cleary, Rachel Kornhaber, Josef Haik, Ayelet Di Segni

**Affiliations:** 1 Sheba Tissue and Cell Bank, Sheba Medical Centre, Tel Hashomer, Israel; 2 National Burn Center, Sheba Medical Center, Tel Hashomer, Israel; 3 The Green Skin Engineering Center, National Burn Center, Sheba Medical Center, Tel Hashomer, Israel; 4 School of Medicine, Tel Aviv University, Tel Aviv, Israel; 5 Talpiot Leadership Program, Sheba Medical Center, Tel Hashomer, Israel; 6 School of Health, University of New England, Armidale, NSW Australia

**Keywords:** Tissue donors, Allografts, Bacterial contamination, Surgical wash

## Abstract

Musculoskeletal allografts are becoming increasingly essential in orthopaedic and reconstructive surgery. A significant source of these allografts is tissues retrieved from deceased donors. While contributing substantially to the pool of allografts, these tissues are at increased risk of bacterial contamination. This risk is further amplified when tissues are procured from field casualties. In this study, we performed a side-by-side retrospective comparison of contamination rates in tissues retrieved from the general population and battlefield mortalities, representing field casualties. In response to the rising incidence of contaminated tissues obtained from field casualty donors, we implemented a more rigorous pre-operative washing protocol designed to maximize sterility. This protocol modification led to a significant decrease in the contamination ratio of tissues retrieved from field casualties. Our findings suggest that current pre-operative cadaver preparation guidelines may need to be reconsidered for tissue donations following unsanitary condition mortalities. Furthermore, these findings strongly suggest that field surgeries would benefit from a surgical pre-operative preparation designed specifically for incidents such as battlefields, accidents or mass casualty incident areas to minimize post-operative infections and improve surgical outcomes.

**Supplementary Information:**

The online version contains supplementary material available at 10.1007/s10561-026-10208-4.

## Introduction

Musculoskeletal (MSK) allografts are increasingly essential in orthopedic and reconstructive surgery for tissue replacement, bone and tendon repairs, and other medical procedures (Della Valle et al. 2024). Given their significant benefits in improving patient outcomes and quality of life, ensuring their safety is crucial. Bacterial contamination can lead to severe clinical consequences, including transmission of infection, graft rejection, and even mortality in transplant recipients (Eastlund 2006; Mroz et al. 2008). Recent bone allograft related tuberculosis outbreaks in the United States exemplify the risk for disease transmission posed by contaminated tissue transplantation (Ruan et al. 2023; Wortham et al. 2024). Consequently, a portion of retrieved tissue may be discarded.

Allografts from deceased donors, which significantly contribute to the pool of available grafts, are particularly prone to microbial contamination (Baseri et al. 2022) due to various inherent factors. Donor bodies may harbor bacteria due to their physiological state at death and environmental exposure before and during the extraction process. To minimize the risk of bacterial contamination, globally accepted standardized guidelines are applied throughout the process, from donor selection and tissue retrieval to processing, storage and distribution (American Association of Tissue Banks 2016; European Directorate for the Quality of Medicines HealthCare 2022).

In this retrospective study, we compare the contamination rates of MSK tissues recovered from two distinct donor populations: the general population and battlefield casualties representing mortalities sustained in field conditions. The study evaluates the impact of an enhanced pre-operative preparation protocol on reducing the elevated tissue contamination observed in battlefield casualty donors. This direct comparison between these two donor populations will guide the development of tailored pre-surgical preparation protocols, particularly in the context of mass casualty events or battlefield mortalities, where contamination risks may be inherently higher. To our knowledge, this is the first study to monitor side-by-side tissue contamination in large cohorts of field casualties and general population donors.

## Materials and methods

### Study design

This study aimed to compare contamination rates in tissue donations from the general population *vs.* field casualty donors and to evaluate the effect of a pre-operative preparation protocol on contamination rates in each population.

To this end, we conducted a retrospective analysis of culture results using a swab method, routinely collected from all donated tissues. The tissues were classified based on the corresponding donor pre-operative preparation protocol employed: (1) standard protocol, utilized from January 2023 to January 2024, and (2) modified protocol implemented starting February 2024, until January 2025. Additionally, within each period, donors were further divided into two groups: (a) members of the general population and (b) field casualties, represented by battlefield mortalities. This retrospective study was conducted under institutional approval from Sheba Medical Center (Helsinki IRB No. 2454–25-SMC-D).

### MSK tissues

Microbiological swab cultures of all MSK tissues retrieved by the Sheba Tissue Bank between January 2023 and January 2025 from 85 donors were analyzed. Table 1 details the number of tissues and donors in each group.

**Table 1:** Number of tissues and donors by group

	Number of donors	Number of tissues
Standard wash, General population	30	501
Standard wash, Field casualties	19	326
Modified wash, General population	26	420
Modified wash, Field casualties	10	158
Total	85	1405

### Recruitment of potential tissue donors and consent

The various stages of the tissue bank activity, from tissue donation to processing and distribution, are regulated by the Ministry of Health (Regulation 36/2013) (Frenkel 2008) and the Israel Transplant Law - Organ Transplantation Act 2008), in accordance with international organizations (American Association of Tissue Banks 2016; European Directorate for the Quality of Medicines HealthCare 2022).

Informed consent is a requirement for tissue donation. Families of the deceased provide their authorization by signing a consent form. Any individual who meets the medical and ethical criteria, and whose family has signed the consent form, can become a donor. In accordance with Israeli Ministry of Health regulations, tissue donations from non-citizens are not permitted.

### Donor eligibility screening process

Tissues are retrieved within 48 h of death if the body was refrigerated within six hours post-mortem, or within 12 h of death if the body was not refrigerated. The donor eligibility screening process for MSK tissues involves three critical steps: communicable disease testing, visual inspection, and assessment of contraindications.

### Communicable disease testing

All potential donors undergo rigorous serological testing to screen for transmissible infections. Blood samples are collected and tested for Human Immunodeficiency Virus (HIV) types 1 and 2, Hepatitis B Virus (HBV), Hepatitis C Virus (HCV), Syphilis, and West Nile virus. Only donors with negative results for all the above tests are eligible.

### Visual inspection

A comprehensive physical examination is performed to identify any physical signs indicative of potential contraindications. In cases involving field casualties, special attention is given to the presence of open fractures or wounds on the limbs. The MSK tissues are visually inspected to ensure their suitability for transplantation. Any tissues exhibiting abnormalities are excluded from further processing and are sent for burial.

### Contraindications

Donors are evaluated for contraindications based on their medical and social history, as outlined in the *Standards for Tissue Banking* by the American Association of Tissue Banks (2016). A detailed review of the donor’s medical records is conducted to identify any history of malignancy, autoimmune disease, or chronic infection that could compromise tissue quality. In addition, the donor's lifestyle is evaluated, including behaviors that might increase the risk of communicable diseases, such as intravenous drug use or risky sexual behavior.

### Surgical pre-operative preparation

Tissue procurement from both the general population and field casualties was performed in hospital operating rooms.

The standard pre-operative preparation procedure was conducted as follows:

1. Surgical washing and scrubbing: Antiseptic agents were applied generously to the donor's body, focusing on the limbs from which tissues will be recovered. The agents were used in a circular motion from the incision site outward to minimize the risk of contamination. The washes were followed by scrubbing with sterile sponges or brushes to enhance the removal of microorganisms. The first wash was performed with a Septal Scrub antiseptic agent containing 4% Chlorhexidine Gluconate. A second wash was performed with an Alcoxidin antiseptic agent containing 0.5% Chlorhexidine Gluconate in 70% alcohol.
2. Draping: After the antiseptic application and once the skin was dried, sterile drapes were applied to cover the body, exposing only the tissue recovery sites.
3. Recovery procedure: Each disinfected limb was covered with Ioban™ drapes to prepare and maintain a sterile field during the recovery procedure.

### Modified surgical pre-operative preparation

In response to the rising incidence of contaminated tissues obtained from field casualty donors who were battlefield fatalities, we implemented an enhanced pre-operative washing protocol designed to maximize decontamination.

The improved protocol comprised the following steps:

1. Both lower limbs were initially washed with Septal Scrub antiseptic solution.
2. The contralateral limb was covered, and the operative limb underwent a second wash before standard antiseptic preparation and draping procedures commenced.
3. Gloves and scalpels were changed frequently throughout the procedure to control cross-contamination.
4. Any scalpel used to incise the skin was replaced before working on internal tissues.
5. In cases where the contralateral limb was deemed unsuitable due to injury, it was thoroughly wrapped in Ioban™ drapes and multiple layers of surgical drapes prior to the commencement of the procedure to ensure maximum isolation.
6. Skin recovery from the limb was prohibited until after the extraction of tendons and bones. The entire limb was considered unsuitable for MSK tissue retrieval if the skin was recovered from a forelimb.

### Microbiological swab cultures

Each tissue is sampled by an eSwab (Copan, Italy, 480CE) directly upon procurement, before packaging in the operating room. The eSwab transport system contains 1 mL of Liquid Amies medium. The swab is transferred to the institutional microbiology facility, where 100 µl of the eSwab transport medium are plated onto each Blood, Chocolate, and Sabouraud Dextrose agar plate, as well as into a Thioglycollate Broth tube, resulting in a total of 400 µl per tissue sample.

### Statistical analysis

The statistical significance of differences between populations and treatment groups was determined using the chi-squared (χ^2^) two-tailed test. The analyses were conducted using an online tool (https://www.standarddeviationcalculator.io/chi-square-calculator).

## Results

### Tissue contamination

During the study’s first phase (January 2023-January 2024), routine microbiological monitoring revealed that 21.5% of the tissues extracted from field casualties were contaminated. This ratio represented a 4.5-fold increase compared to tissues extracted from the general population, which presented 4.6% contamination.

**Fig. 1:**
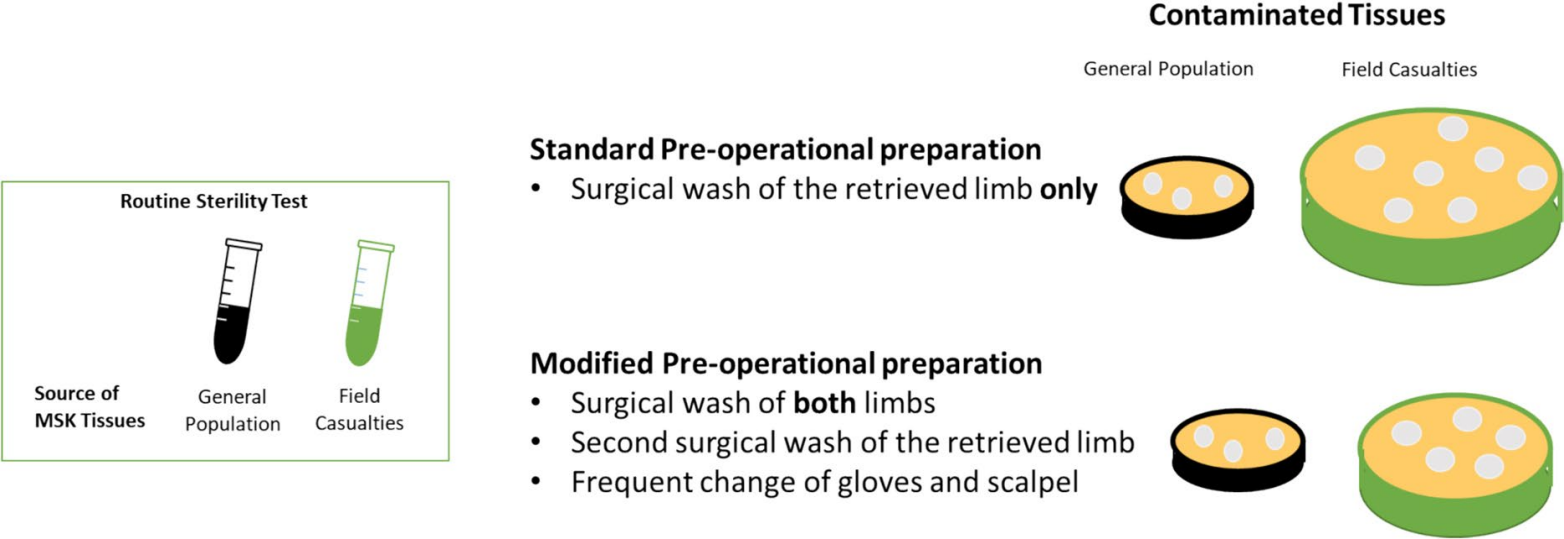
Study outline: Comparison of MSK tissue contamination rates between field casualty and general population donor groups, implementing standard and modified rigorous cadaver pre-operative preparation protocol. Under standard preparation protocol, field casualty donors demonstrated significantly higher contamination rates (with rates represented by Petri dish size), which were substantially reduced by implementing the modified pre-operative preparation protocol.

A modified pre-operative protocol was implemented to reduce contamination rates, which included surgical preparation of both limbs, covering of the contralateral limb, and a second wash of the operative limb. Additionally, frequent scalpel and glove changes were applied (Fig. 1).

**Fig. 2:**
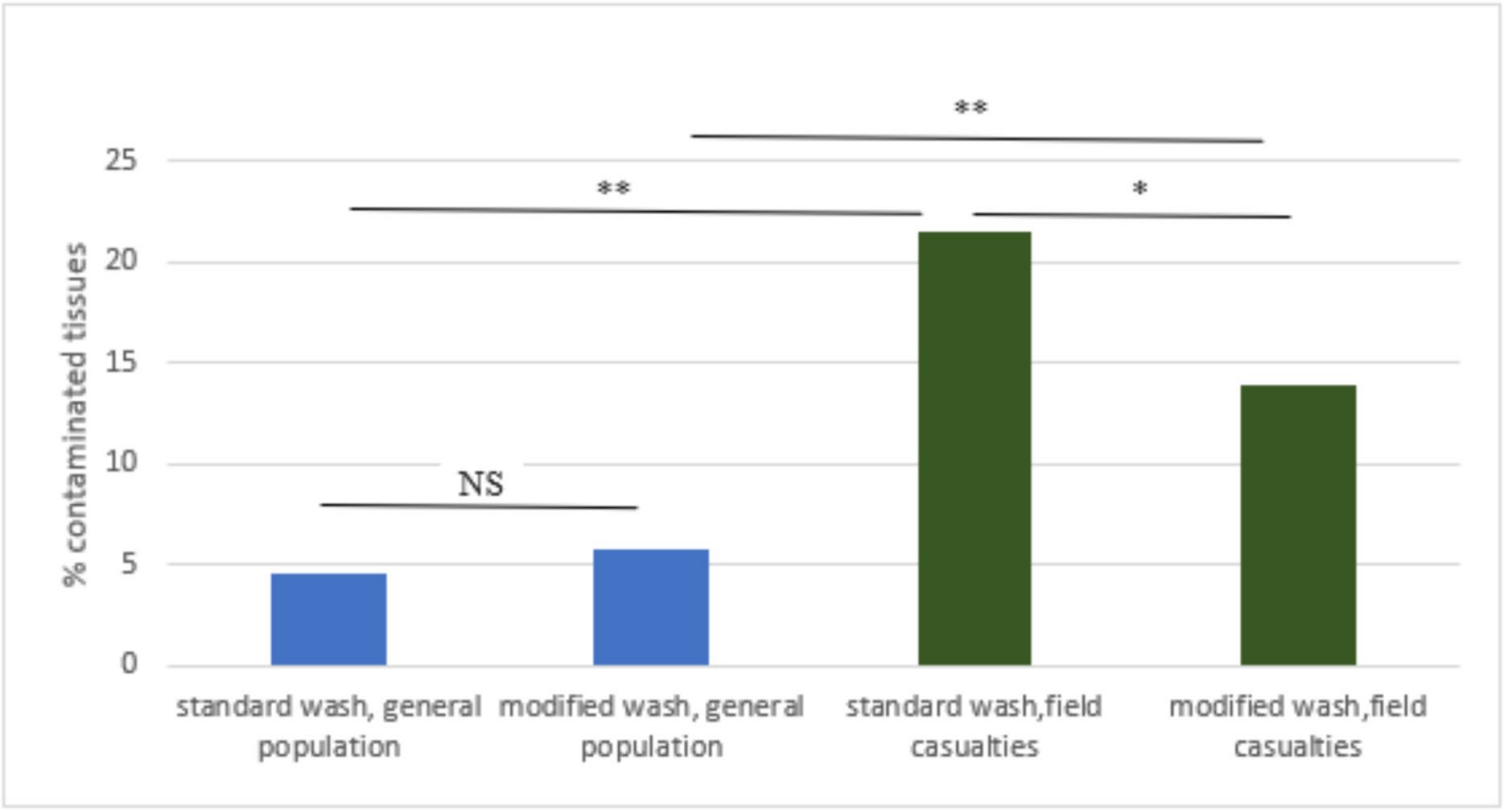
Frequency of contaminated MSK tissues

Following this modification, a significant decrease in the rate of contaminated tissues extracted from field casualties to 13.9% was observed. In tissues extracted from deceased donors in the general population, a 5.7% contamination rate was not significantly different compared to tissues extracted with a standard wash (Fig. 2).

MSK tissues recovered from the general population or field casualties following standard or modified pre-operative preparation were routinely screened for bacterial contamination. The ratio of contaminated tissues represents the percentage of contaminated out of the total tissues recovered from each group. **P-value < 0.001; *P-value ≤ 0.05; NS—not significant.

### Contaminating bacteria

**Fig. 3:**
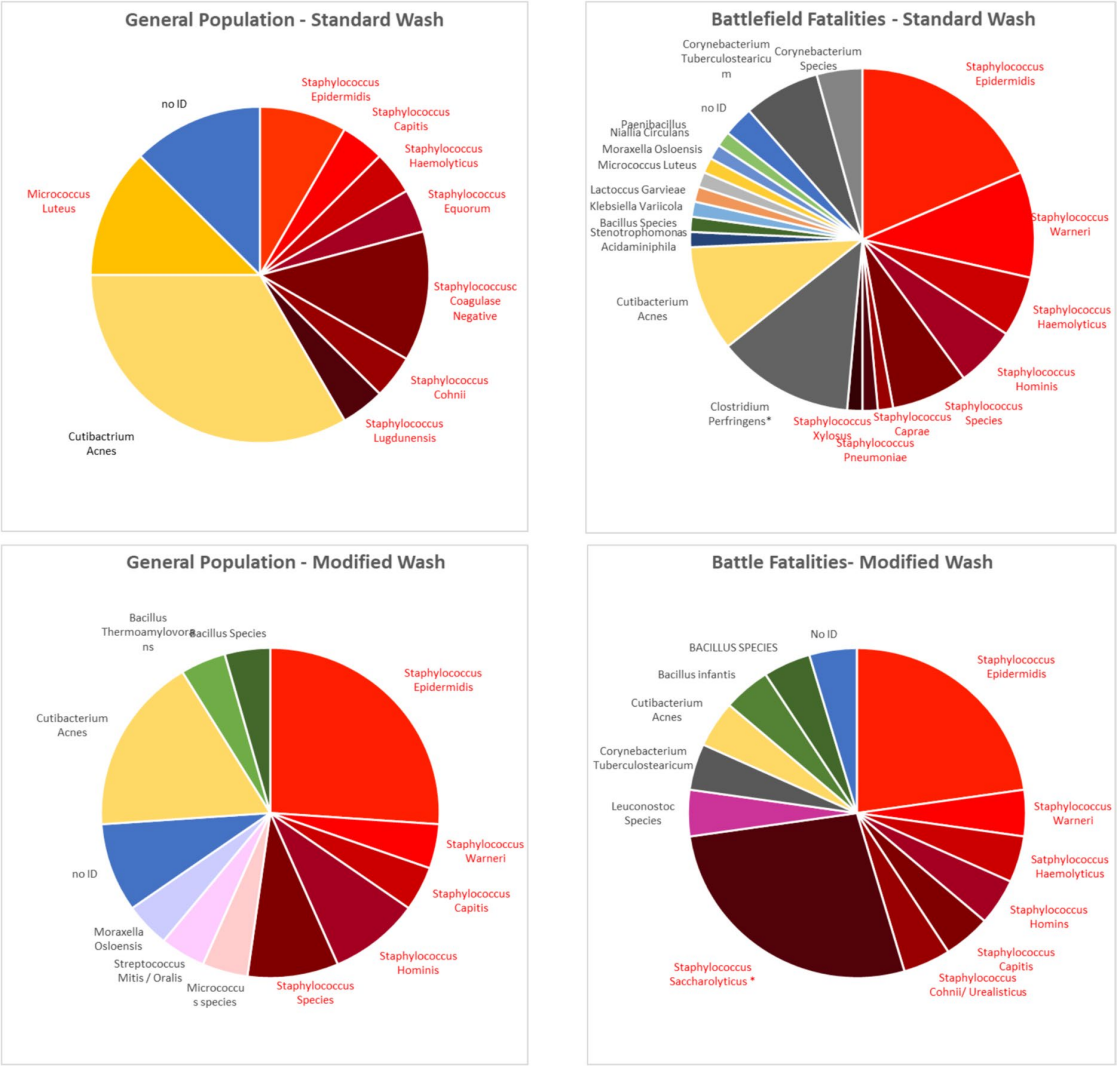
Distribution of contaminating bacterial species

Following standard pre-operative washing, contaminated tissues extracted from field casualties presented a high diversity in bacterial strains, as 18 different strains were identified in this population (excluding “no ID” or no genus identified; Fig. 3, Table S1). This high diversity decreased to eleven different strains following the modification of the cadaver washing protocol (Fig. 3 & Table S2).

The distribution of bacterial species identified in each donor's population and wash protocol is presented. Asterisks indicate bacteria found in a single donor only. Staphylococci are shown in shades of red. In the general population, the diversity of bacterial strains (as well as contamination rates) was consistently lower than that of the field casualties, with nine and eight strains identified before and after changing the preparation protocol, respectively (Tables S1, S2).

**Fig. 4:**
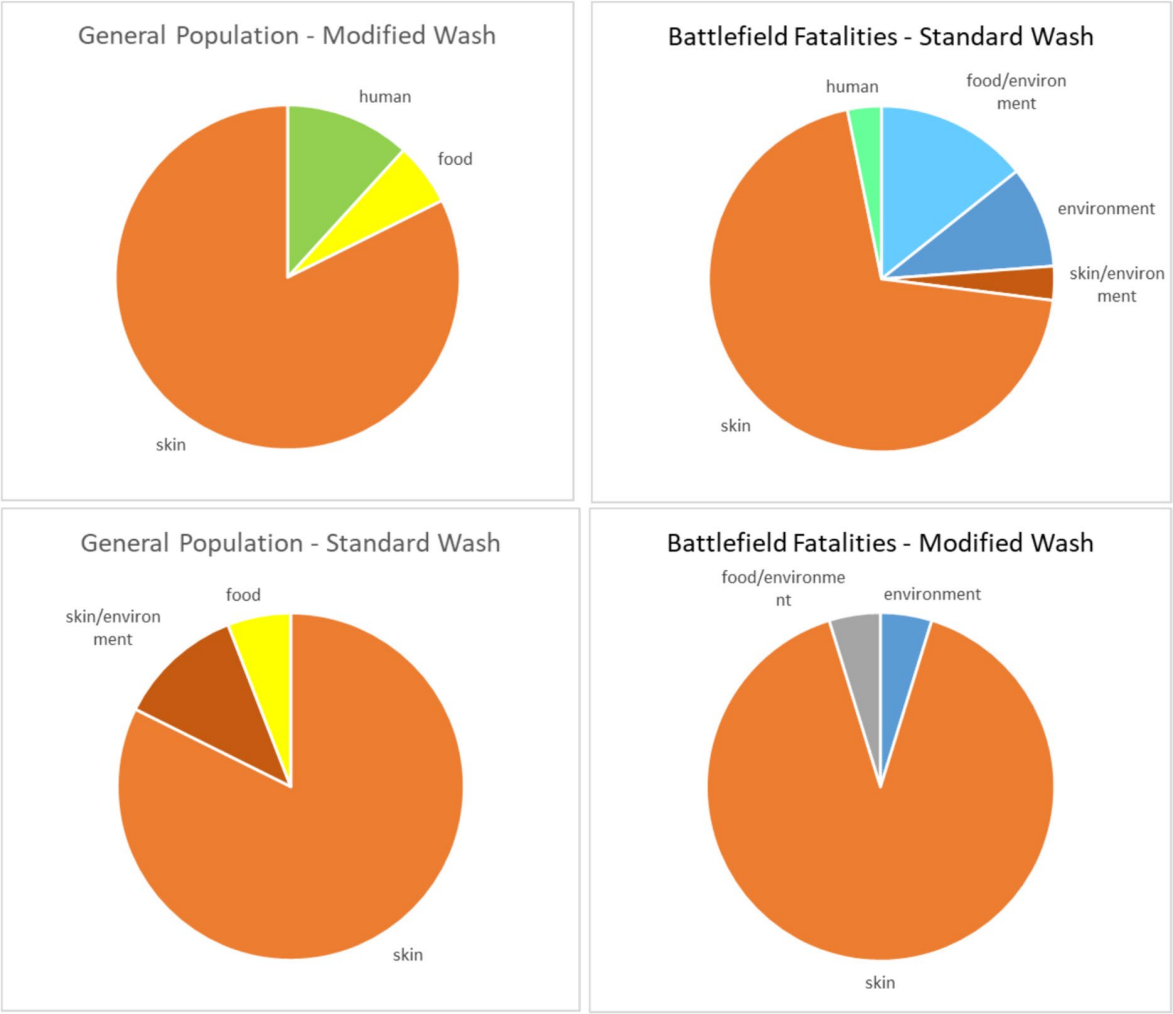
Distribution of contamination source. Bacterial species with defined identification were divided according to their most likely source

In both the general population and field casualty groups, and independent of the washing protocol, most contaminating bacterial strains belonged to the skin microbiota, accounting for more than 70% of contaminations with definitive identification (Fig. 4 & Tables S1, S2). The main bacterial species were staphylococci, accounting for 42–72% of the contaminating bacteria, dominated mainly by *Staphylococcus epidermidis*, a part of the healthy human epithelial microbiota.

Notably, changing the cadaver preparation protocol removed most low-prevalence species while elevating the relative representation of staphylococci. Interestingly, *Cutibacterium acnes*, another skin bacterium that was prevalent in 10–35% of the contaminated tissues procured following standard wash, was reduced by 50% in both populations, to 4.5% and 17%, respectively (Fig. 3 & Tables S1, S2). These observations indicate that MSK tissue contamination predominantly resulted from skin microbiota. Tissues retrieved from field donors are prone to higher contamination rates, which can be substantially decreased with the implementation of a rigorous pre-operative preparation protocol.

## Discussion

Bacterial contamination of bone and tendon allografts poses a serious health hazard to potential allograft recipients and leads to the disposal of a significant portion of donated tissue. Tissues extracted from cadaver donors, while being a significant source of these allografts, are at an increased risk of contamination (Baseri et al. 2022). This risk is further augmented when tissues are procured from field casualties such as mass casualties and battlefield mortalities: The environmental conditions increasing the exposure to pathogens; extended time interval between death and tissue retrieval, delayed cadaver refrigeration; and potentially the mode of death (*e.g*., trauma) are all associated with heightened risk for bacterial contamination (Centers for Disease Control and Prevention (CDC) 2002; Brubaker et al. 2016; Schubert et al. 2012).

Reported bacterial contamination rates of MSK allografts sampled at the time of retrieval vary widely across studies, ranging from approximately 6% to nearly 60% (Brubaker et al. 2016; Schubert et al. 2012; Paolin et al. 2017; Ireland and Spelman 2005; Varettas 2013; Segur et al. 2000; Forsell and Liesman 2000; Ilays et al. 2021; Ibrahim et al. 2004). In this study, we compared bacterial contamination rates in MSK tissues extracted from either the general population or from field casualties. In the general population, applying globally accepted cadaver washing guidelines, we found bacterial contamination at a rate similar to the lower reported range of around 6%. In contrast, for field casualties, the same retrieval skin decontamination protocol was less effective, as reflected by higher contamination rates observed in recovered MSK tissues.

In both the field casualty and general population groups, the contaminating bacterial species were predominantly skin commensals, in accordance with previous reports of tissue bank contaminations (Brubaker et al. 2016; Schubert et al. 2012; Paolin et al. 2017; Ireland and Spelman 2005; Varettas 2013; Segur et al. 2000; Ilays et al. 2021; Ibrahim et al. 2004), as well as battlefield wound infections (Staruch and Hettiaratchy 2019). A systematic review and meta-analysis (Baseri et al. 2022) identified *Staphylococcus* spp. as the predominant contaminant, followed by *Propionibacterium* spp. Accordingly, we found that more than 50% of the contaminating bacteria are staphylococci, primarily Staphylococcus epidermidis, which form part of the skin's normal microflora. *Cutibacterium acnes* (formerly *Proprionibacterium acnes*), another member of the skin microbiota, was also consistently present in a large ratio of the contaminated tissues. These observations suggest pre-existing bioburden derived from the native microbiota present in or on the donor’s tissues prior to recovery. This aligns with the concept of endogenous contamination, as previously described in the literature (Ibrahim et al. 2004). Alternatively, personnel-related or surgical instrument contamination during tissue retrieval is a plausible explanation, potentially implicating the retrieval process itself and highlighting the role of personnel and surgical instruments in contributing to MSK tissue contamination.

Recently, the PREP-IT Investigators (Investigators et al. 2024) demonstrated that a pre-operative skin antisepsis protocol using iodine povacrylex is associated with fewer surgical site infections compared to chlorhexidine gluconate. Consequently, after finding elevated contamination rates among field casualty donors, we decided to modify the donors' surgical preparation guidelines to include a more rigorous washing process for the entire cadaveric donor, rather than only the procured donor site. In addition, to minimize tissue infections by skin microflora, the most frequent contamination source, we avoid taking skin before tissue extraction from the same limb. Implementing these precautions and a stringent washing protocol resulted in a significant reduction in tissue contamination among field casualties; however, in the general population's tissues, contamination rates were unaffected.

The modified preparation guideline removed low-frequency contaminants from both general and soldier populations, decreasing the diversity of bacterial species while leaving the proportion of the more prevalent bacteria. Notably, after modifying the preparation guidelines, the contamination ratio in tissues from field casualties remained significantly higher than that in the general population. This difference is potentially attributable to the extreme battlefield conditions and trauma-associated causes of death. Additionally, factors such as cadaver refrigeration and the length of time from death to tissue extraction, although maintained within recommended guidelines, may approach the limits of permissible timelines, which cannot be fully standardized in field casualty scenarios and may exacerbate contamination risks.

During this study, the Sheba Tissue and Cell Bank treated soldiers admitted to the Sheba Burn Center with cultured epithelial autografts (CEA). These soldiers suffered from severe trauma, including extensive burns. Consistent with our findings, we observed increasingly high contamination rates in the cultured grafts, which had to be subsequently discarded (data not shown). We hypothesize that implementing the proposed improved surgical preparation guidelines before biopsy collection has the potential to reduce the initial skin bioburden and the associated incidence of CEA culture contaminations.

This study presents the first panel to include a side-by-side comparison of MSK tissue contamination between field casualty and general population donors. Our findings demonstrate that field donors have a significantly higher ratio of contaminated tissues, which can be reduced by employing a modified, more stringent surgical preparation protocol. Our findings suggest that current guidelines for the preparation of tissue donors may need to be reconsidered to effectively reduce bacterial contamination in donors with traumatic causes of death, particularly those with extensive penetrating wounds.

## Conclusion

The implications of our results are particularly significant for field surgeries conducted in war or mass casualty incident zones. In such scenarios, the risk of contamination is significantly elevated, and the availability of sterile surgical environments is often limited. It is important to clarify that all tissues included in the present study were procured in hospital operating rooms under strictly controlled aseptic conditions, with adherence to established sterility protocols. Nonetheless, we posit that in scenarios where sterile environments cannot be fully maintained, such as field surgeries, meticulous surgical pre-operative preparation becomes indispensable to mitigate contamination risks and preserve tissue integrity. In addition, this modification may also be relevant in cases of surgeries following trauma with open fractures or penetrating wounds, such as those sustained in severe car accidents. Adopting enhanced surgical preparation protocols in these settings is crucial, as it will promote higher standards of care and substantially minimize the risk of post-operative infections, particularly surgical wound infections. Furthermore, implementing these stringent guidelines will likely reduce the need for antibiotic treatment among potential allograft recipients mitigating the potential for drug resistance. We believe these considerations can potentially enhance surgical outcomes and ultimately reduce the risk of disease transmission associated with MSK tissue transplantation.

## Supplementary Information

Below is the link to the electronic supplementary material.

Supplementary file1 (DOCX 17 kb)

## Data Availability

The data that support the findings of this study are not openly available due to reasons of sensitivity and are available from the corresponding author upon reasonable request.

## References

[CR19] American Association of Tissue Banks (2016) Standards for Tissue Banking. 14 ed. Maryland. https://www.aatb.org/standards

[CR1] Baseri N, Meysamie A, Campanile F, Hamidieh AA, Jafarian A (2022) Bacterial contamination of bone allografts in the tissue banks: a systematic review and meta-analysis. J Hosp Infect 123:156–173. 10.1016/j.jhin.2021.10.020 34752801 10.1016/j.jhin.2021.10.020

[CR2] Brubaker S, Lotherington K, Zhao J, Hamilton B, Rockl G, Duong A, Garibaldi A, Simunovic N, Alsop D, Dao D, Bessemer R, Ayeni OR, Bioburden Steering Committee and Tissue Recovery Working Group (2016) Tissue recovery practices and bioburden: a systematic review. Cell Tissue Bank 17(4):561–571. 10.1007/s10561-016-9590-5 27761677 10.1007/s10561-016-9590-5 PMC5116036

[CR3] Centers for Disease Control and Prevention (CDC) (2002) Update: allograft-associated bacterial infections–United States, 2002. MMWR Morb Mortal Wkly Rep 51(10):207–210 11922189

[CR4] Della Valle A, Compagnoni R, Puglia F et al (2024) Allografts use in orthopedic surgery: trend change over the past 11 years from a regional tissue bank. Cell Tissue Bank 25(2):713–720. 10.1007/s10561-024-10134-3 38386210 10.1007/s10561-024-10134-3

[CR5] Eastlund T (2006) Bacterial infection transmitted by human tissue allograft transplantation. Cell Tissue Bank 7(3):147–166. 10.1007/s10561-006-0003-z 16933037 10.1007/s10561-006-0003-z

[CR6] European Directorate for the Quality of Medicines & HealthCare (2022) Guide to the quality and safety of tissues and cells for human application. 5 ed. France: European Committee on Organ Transplantation. https://cnrha.sanidad.gob.es/documentacion/bioetica/pdf/guide-to-the-quality-and-safety-of-tissues-and-cells-for-human-application-5th-edi.PDF Accessed 2 December 2024

[CR7] Forsell JH, Liesman J (2000) Analysis of potential causes of positive microbiological cultures in tissue donors. Cell Tissue Bank 1(2):111–115. 10.1023/A:1010106214542 15256955 10.1023/A:1010106214542

[CR8] Frenkel DA (2008) Organ transplants in Israel under the anatomy and pathology law. Harefuah 147(5):413–416 18770963

[CR9] Ibrahim T, Stafford H, Esler CN, Power RA (2004) Cadaveric allograft microbiology. Int Orthop 28(5):315–318. 10.1007/s00264-004-0579-5 15480661 10.1007/s00264-004-0579-5 PMC3456985

[CR10] Ilays I, Alsakran SA, Fallatah AB, Alyateem M, Al-Mohrej OA (2021) The contamination of allografts in multi-organ donors: a bone bank experience. Cell Tissue Bank 22(3):499–504. 10.1007/s10561-020-09899-0 33420876 10.1007/s10561-020-09899-0

[CR11] Ireland L, Spelman D (2005) Bacterial contamination of tissue allografts - experiences of the donor tissue bank of Victoria. Cell Tissue Bank 6(3):181–189. 10.1007/s10561-005-7365-5 16151958 10.1007/s10561-005-7365-5

[CR12] Israel Transplant Law - Organ Transplantation Act 2008 (2008). https://sections.tts.org/DOI/Israel%20Transplant%20Law.pdf

[CR13] Mroz TE, Joyce MJ, Steinmetz MP, Lieberman IH, Wang JC (2008) Musculoskeletal allograft risks and recalls in the United States. J Am Acad Orthop Surg 16(10):559–565. 10.5435/00124635-200810000-00001 18832599 10.5435/00124635-200810000-00001

[CR14] Paolin A, Trojan D, Petit P, Coato P, Rigoli R (2017) Evaluation of allograft contamination and decontamination at the Treviso Tissue Bank Foundation: a retrospective study of 11,129 tissues. PLoS ONE 12(3):e0173154. 10.1371/journal.pone.0173154 28267776 10.1371/journal.pone.0173154 PMC5340366

[CR15] PREP-IT Investigators, Sprague S, Slobogean G et al (2024) Skin antisepsis before surgical fixation of extremity fractures. N Engl J Med 390(5):409–420. 10.1056/NEJMoa2307679 38294973 10.1056/NEJMoa2307679

[CR16] Ruan T, Naveed M, Vien H (2023) Case report: tuberculosis recall on bone graft patient. N Am Spine Soc J 15:100241. 10.1016/j.xnsj.2023.100241 37483264 10.1016/j.xnsj.2023.100241 PMC10362344

[CR17] Schubert T, Bigaré E, Van Isacker T, Gigi J, Delloye C, Cornu O (2012) Analysis of predisposing factors for contamination of bone and tendon allografts. Cell Tissue Bank 13(3):421–429. 10.1007/s10561-011-9291-z 22212704 10.1007/s10561-011-9291-z

[CR18] Segur JM, Suso S, García S, Combalía A, Fariñas O, Llovera A (2000) The procurement team as a factor of bone allograft contamination. Cell Tissue Bank 1(2):117–119. 10.1023/A:1010136931919 15256956 10.1023/A:1010136931919

[CR20] Staruch RMT, Hettiaratchy S (2019) Warzone trauma and surgical infections. Surg Infect (Larchmt) 37(1):58–63. 10.1016/j.mpsur.2018.12.001

[CR21] Varettas K (2013) Micro-organisms isolated from cadaveric samples of allograft musculoskeletal tissue. Cell Tissue Bank 14(4):621–625. 10.1007/s10561-013-9363-3 23340929 10.1007/s10561-013-9363-3

[CR22] Wortham JM, Haddad MB, Stewart RJ et al (2024) Second nationwide tuberculosis outbreak caused by bone allografts containing live cells - United States, 2023. MMWR Morb Mortal Wkly Rep 72(5253):1385–1389. 10.15585/mmwr.mm725253a1 38175804 10.15585/mmwr.mm725253a1

