# Supplementary material for: Enhancing cadaver preparation protocol to reduce bacterial contamination in musculoskeletal allografts: a comparative study of battlefield fatalities and general population donors
Source: Cell Tissue Bank. 2026 Feb 25;27(1):14. doi: 10.1007/s10561-026-10208-4 (PMC12935800; doi:10.1007/s10561-026-10208-4)
Supplement: Supplementary file 1 — Supplementary file1 (DOCX 17 kb) [file 10561_2026_10208_MOESM1_ESM.docx]

**Table S1** Classification of bacteria identified in contaminated tissues procured using the standard preparation protocol with respect to their natural sources.

|  | **Standard Wash** | **General Population** | **Field Casualties** |
| --- | --- | --- | --- |
| **Source** | **Bacteria** | **# of contaminated tissues** | **# of contaminated tissues** |
| Skin | Corynebacterium tuberculostearicum | 0 | 5 (single donor) |
|  | Cutibacterium acnes | 8 | 7 |
|  | Staphylococcus capitis | 1 | 0 |
|  | Staphylococcus caprae | 0 | 1 |
|  | Staphylococcus cohnii | 1 | 0 |
|  | Staphylococcus epidermidis / lugdunensis | 0 | 1 |
|  | Staphylococcus epidermidis / haemolyticus / warneri | 0 | 1 |
|  | Staphylococcus epidermidis | 2 | 13 |
|  | Staphylococcus haemolyticus | 1 | 4 |
|  | Staphylococcus hominis | 0 | 4 |
|  | Staphylococcus hominis/species | 0 | 1 |
|  | Staphylococcus lugdunensis | 1 | 0 |
|  | Staphylococcus warneri | 0 | 7 |
|  | Gram positive, no identification | 1 | 1 |
|  | Corynebacterium species | 0 | 3 |
|  | Staphylococcus species | 0 | 2 |
|  | Staphylococcus, Coagulase negative | 3 | 0 |
|  |  |  |  |
| Environmental and skin | Micrococcus luteus | 2 | 1 |
|  | Staphylococcus xylosus | 0 | 1 |
|  |  |  |  |
| Environmental | Klebsiella variicola | 0 | 1 |
|  | Lactoccus garvieae | 0 | 1 |
|  | Bacillus species | 0 | 1 |
|  | Niallia circulans | 0 | 1 |
|  | Paenibacillus amylolyticis | 0 | 1 |
|  | Stenotrophomonas acidaminiphila | 0 | 1 |
|  |  |  |  |
| Environmental and food | Clostridium perfringens | 0 | 9 (single donor) |
|  | Staphylococcus equorum | 1 | 0 |
|  |  |  |  |
| Human respiratory tract | Moraxella osloensis | 0 | 1 |
|  | Staphylococcus pneumoniae | 0 | 1 |
|  |  |  |  |
| N/A* | Gram negative rod | 0 | 1 |
|  | no identification | 2 | 0 |

N/A*: contamination without a definite identification of bacteria. Source could not be allocated.

**Table S2** Classification of bacteria identified in contaminated tissues procured with modified preparation protocol with respect to their natural sources.

|  | **Modified (stringent) wash** | **General population** | **Field casualties** |
| --- | --- | --- | --- |
| **Source** | **Bacteria** | **# of contaminated tissues** | **# of contaminated tissues** |
| Skin | Corynebacterium tuberculostearicum | 0 | 1 |
|  | Cutibacterium acnes | 4 | 1 |
|  | Satphylococcus haemolyticus | 0 | 1 |
|  | Staphylococcus capitis | 1 | 1 |
|  | Staphylococcus epidermidis | 6 | 5 |
|  | Staphylococcus hominis | 2 | 1 |
|  | Staphylococcus warneri | 1 | 1 |
|  | Staphylococcus Saccharolyticus | 0 | 6 (single donor) |
|  | Staphylococcus cohnii/ urealyticus | 0 | 1 |
|  | Staphylococcus epidermitis & hominis / cutibacterium acnes | 0 | 1 |
|  | Micrococcus species | 1 | 0 |
|  | Staphylococcus species | 2 | 0 |
|  | Gram positive | 2 | 0 |
|  |  |  |  |
| Environmental | Bacillus infantis | 0 | 1 |
|  | Bacillus species | 1 | 1 |
|  |  |  |  |
| Environmental and food | Leuconostoc species | 0 | 1 |
|  | Bacillus thermoamylovorans | 1 | 0 |
|  |  |  |  |
| Human respiratory tract | Moraxella osloensis | 1 | 0 |
| Human oral cavity | Streptococcus mitis / oralis | 1 | 0 |
|  |  |  |  |
| N/A* | Gram variable rod | 1 | 0 |

N/A*: contamination without a definite identification of bacteria. Source could not be allocated.
